# Healthcare workers knowledge and diagnostic practices: a need for dengue and chikungunya training in Moshi Municipality, Kilimanjaro Tanzania

**DOI:** 10.1186/s13104-019-4074-x

**Published:** 2019-01-18

**Authors:** Samwel Saringe, Debora C. Kajeguka, Dickson D. Kagirwa, Maseke R. Mgabo, Basiliana Emidi

**Affiliations:** 1 Kilimanjaro Christian Medical University College, P.O. Box 2240, Moshi, Tanzania; 2 National Institute for Medical Research, Headquarters, P.O. Box 9653, Dar es Salaam, Tanzania; 3 Institute of Rural Development Planning, P.O. Box 138, Dodoma, Tanzania

**Keywords:** Dengue, Chikungunya, Knowledge, Diagnostic practices, Tanzania

## Abstract

**Objective:**

Dengue and chikungunya virus diseases are becoming an increasingly important global health threats and are continuously expanding their geographical range. The study aims to investigate knowledge and diagnostic practice of dengue and chikungunya fever among healthcare workers in Moshi Municipality.

**Results:**

Most of healthcare workers heard of chikungunya and dengue 146 (71.2%) and 203 (99%) respectively. Ninety-five (46.3%) and 152 (74.1%) had good knowledge regard chikungunya and dengue respectively. One hundred and twenty-two of HCWs 122 (59.5%) reported that there is no vaccination for dengue virus. Most HCWs 199 (97.0%) reported that the absence of diagnostic tool for dengue virus lead to difficult in managing the infection. The finding of this study showed that there is insufficient knowledge regarding chikungunya while knowledge regarding dengue is relatively fair. This calls for training regarding these infections.

**Electronic supplementary material:**

The online version of this article (10.1186/s13104-019-4074-x) contains supplementary material, which is available to authorized users.

## Introduction

Chikungunya and dengue are currently among the important Arbovirus. Chikungunya fever is caused by the chikungunya virus of the family *Togaviridae* and genus *Alphavirus* and dengue fever is caused by the virus of the genus *Flaviviru*s the family *Flaviviridae*. Both infections are transmitted between human by the bite of infected *Aedes aegypti* and to a less extent *Aedes albopictus* [1–3]. Dengue has rapidly spread in all World Health Organization (WHO) regions in recent years [4]. It is estimated that over 50% of the world’s population is at risk of dengue infection [4], with prediction of almost 400 million dengue infections occurring each year [5]. Notwithstanding increasing reports of chikungunya infections in different parts of the world, detailed information on the global population at risk remains sparse, however it is estimated that 1.5 billion of the world population is at risk of chikungunya infection [6], with 2 million infections annually [7].

Given the time when dengue-like illness were detected, one would expect adequate knowledge on diagnostic practices of this disease among health care workers (HCWs). However, studies indicate that understanding and diagnosing dengue vary considerably. For example, a study done in government and private hospitals as well as clinics in Karachi indicated that 100% of the doctors were aware about dengue viral infection but lack knowledge about its diagnosis (72%) and management, while 50% of doctors wanted to isolate the patient [8]. Another study reported that a large number of physicians lacked knowledge of the probable diagnosis of dengue and the appropriate time to discharge the patients [9]. A recent systematic review found that risk perceptions, attitudes, and knowledge of chikungunya among the public and health professionals vary across populations and countries and knowledge is higher in areas that have experienced an outbreak [10].

Although dengue and chikungunya are reported to prevail in Tanzania [11–14], challenges in diagnosing dengue and chikungunya are still reported in Tanzania [13, 15–18]. Previous research focused on status of exposure to dengue and chikungunya viruses; however, there have been few attempts to date to better understand the knowledge, attitude and practices among HCWs. These studies have been conducted mainly in Kilosa Morogoro [19] and Hai Kilimanjaro [20]; to our knowledge, no studies to date have been conducted in Moshi, Kilimanjaro.

Tanzania has seen a surge of dengue infections since 2010, therefore, there is increasing need for skilled HCWs in managing dengue. Understanding how HCWs manage suspected cases of dengue is crucial to improving patient outcomes. HCWs who interact directly with patients have an important role in both treating and preventing the spread of dengue [21]. Therefore, the aim of this study was to determine knowledge and diagnostic practices regarding dengue and chikungunya among HCW in health facilities in Moshi Municipality.

## Main text

### Material methods

A descriptive cross sectional was conducted in Moshi Municipality in Kilimanjaro region from March to May 2017. Moshi municipality is one of the districts in Kilimanjaro region located at the lower slopes of Mount Kilimanjaro in Northern part of Tanzania. There are 56 health facilities in Moshi Municipal. A total of 15-health facilities both public and private were randomly selected. Public health facilities are Bondeni dispensary, Chuo Cha Polisi health centre, Majengo health centre, Njoro dispensary, Shirimatunda dispensary, Pasua Health centre, Rau dispensary and Mawenzi hospital. Private health facilities are Mary land health centre, Moshi-Arusha hospital, Shanti Town Dispensary, St Joseph hospital, Upendo Health centre, YMCA dispensary and CRCT Kilimanjaro First Health.

The sampling technique for this study was non-probability convenience sampling. In this technique the HCWs that were present in particular health facility at the time of data collection were interviewed after consultation with the head of heath facility.

Face to face interviews were conducted using structured questionnaires with questions specifically designed for HCWs. Interviews were conducted in English and Swahili language.

The study instrument was developed following an extensive review of the literature. The questionnaire was pretested among two separate groups of 5 residents in Kilimanjaro Christian Medical University College and nurses who were excluded from the main study. Inputs from the residents were then used to refine the questionnaire. The questionnaire covered the following areas: (1) demographic information (facility, sex, age and occupation), (2) health information relating to whether the respondent had heard chikungunya and dengue disease or not, (3) knowledge about chikungunya and dengue symptoms, signs, and transmission modes, (4) diagnosis and treatment of chikungunya and dengue.

Knowledge of dengue and chikungunya was quantified using knowledge score as described by Itral et al. and Al-zurfi et al. with few modifications [22, 23]. For HCWs, good knowledge was assessed as participants answered questions correctly pertaining to signs, symptoms, and diagnostic practices for dengue and chikungunya. Correct answers for knowledge item were coded as “10” while incorrect answers were coded as “0”. The total knowledge ranged from 0 to 100 with scores of ≥ 40 or higher being considered as “good” and < 40 and below being considered as “poor”.

### Statistical analyses

Data were analysed using Statistical Package for Social Sciences 20.0 software (SPSS Inc., Chicago, USA). Descriptive statistics are presented as proportions for categorical data. Mean knowledge score was calculated using student *t* test. Univariate analysis was performed by logistic regression. A significance level of ≤ 0.05 was used throughout.

### Results

#### Socio-demographic characteristics of studied population

A total of 205 HCWs were included in the study. Half of the HCWs were from the health centres (105/205: 51.2%), while majority (128/205; 62.4%) were male. A hundred and five (51.2%) were aged between 31 and 40 years, (82/205:40.0%) were nurses. More than half of HCWs (112/205: 54.6%) had a work experience of 5 to 10 years.

#### Knowledge and treatment regarding chikungunya and dengue

Of 205 HCWs, majority (146/205; 71.2%) had heard chikungunya fever virus, and among these, only (85/205; 41.5%) heard through other people (such as friends and neighbours). Few HCWs (80/205; 39.0%) reported that chikungunya is caused by virus while others (44/205; 21.0%) wrongly reported that chikungunya is caused by parasites. Seventy-six (37.1%) of the HCWs answered that the main symptom of chikungunya is muscle pain and almost all HCWs (192/205; 93.7%) reported that not all mosquitoes transmit chikungunya. Eighty-five (41.8%) reported the vector that transmitting this virus is *Aedes* mosquito and (57/205; 27.8%) knew that the infection is transmitted through *Culex* mosquito. Few participants (38/205; 18.5%), knew that the mosquito which chikungunya virus bite during the day time. A number of the HCWs correctly reported that chikungunya virus infections are transmitted by the same vector as yellow fever virus, (40/205; 19.5%) while (37/205; 18.1%) correctly answered that chikungunya virus infections are transmitted by the same vector as Zika virus. About half of HCWs knew that the drug that is used to treat patients infected with chikungunya virus is Anti-pain (102/205; 49.8%) Table 1.

**Table 1:** Knowledge regarding chikungunya and dengue infections among healthcare workers

Variable	Chikungunya (N = 205)	Dengue (N = 205)
	n (%)	n (%)
*Have you heard about these infections?*		
Yes	146 (71.2)	203 (99.0)
No	59 (28.8)	2 (1.0)
*Where did you heard of (infections)?*		
Media	31 (15.1)	138 (67.3)
Books	28 (13.7)	38 (18.5)
People	85 (41.5)	27 (13.2)
Others^a^	2 (1.0)	0 (0.0)
Never heard	59 (28.8)	2 (1.0)
*What is the causative agent?*		
Fungi	2 (1.0)	0 (0.0)
Bacteria	19 (9.3)	17 (8.3)
Virus	80 (39.0)	106 (51.7)
Parasites	44 (21.0)	80 (39.0)
Don’t know	60 (29.3)	2 (1.0)
*What are the symptoms of an infection?*		
Muscle pain	76 (37.1)	116 (56.6)
Vomiting	45 (21.9)	53 (25.9)
Diarrhoea	23 (11.2)	31 (15.1)
All	2 (1.0)	3 (1.5)
Don’t know	59 (28.8)	2 (1.0)
*Are all mosquitoes transmitting infection?*		
Yes	3 (1.5)	3 (1.5)
No	192 (93.7)	201 (98.0)
Don’t know	10 (4.9)	1 (0.5)
*Way of transmit infection*		
*Anopheles*	3 (1.5)	8 (3.9)
*Culex*	57 (27.8)	64 (31.2)
*Aedes*Mosquitoes	85 (41.8)	130 (63.4)
Don’t know	60 (29.3)	3 (1.5)
*At what time do the mosquitoes that transmit these infections bite?*		
Night time	11 (5.4)	9 (4.4)
Day time	38 (18.5)	69 (33.7)
Both day and night	81 (39.5)	102 (49.8)
Don’t know	75 (36.6)	25 (12.3)
*Which virus/parasite is transmitted by the same vector as chikungunya or/dengue*		
Yellow fever virus	40 (19.5)	52 (25.4)
Malaria parasite	13 (6.4)	12 (5.9)
Zika virus	37 (18.1)	63 (30.7)
Rift valley fever virus	25 (12.2)	37 (18.0)
Don’t know	57 (27.7)	4 (2.0)
*A patient with which types of symptoms are supposed to be admitted in hospital?*		
With severe symptoms	52 (25.4)	85 (41.4)
Without symptoms	108 (52.6)	110 (53.7)
Don’t know	3 (1.5)	7 (3.4)
*What drug(s) are used for treatment*		
Anti-pain	102 (49.8)	151 (73.7)
Antimalarial	10 (4.9)	10 (4.9)
Antiviral	35 (17.1)	38 (18.5)
Don’t know	58 (28.3)	6 (2.9)

^a^Such as internet

Almost all HCWs (203/205; 99.0%) heard dengue fever, among these (138/205; 67.3%) heard through media. Half of HCWs (106/205; 51.7%) correctly reported that dengue fever is caused by virus while (80/205; 39.0%) wrongly reported that dengue fever is caused by parasites. One hundred and sixteen (56.6%) of the HCWs answered that the main symptom of dengue fever is muscle pain and almost all HCWs (201/205; 98.0%) reported that not all mosquitoes transmit dengue virus. More than half (130/205; 63.4%) correctly reported that *Aedes* mosquito transmit dengue while (64/205; 31.2%) wrongly reported that the infection is transmitted through *Culex* mosquito. Few HCWs (69/205; 33.7%), knew that the mosquito which transmit dengue virus bite during the day time. A number of the HCWs correctly reported that dengue virus infections are transmitted by the same vector as yellow fever virus, (52/205; 25.4%) while (63/205; 30.7%) correctly answered that dengue virus infections are transmitted by the same vector as Zika virus. More than half of HCWs knew that the drug that is used to treat patients infected with dengue virus is Anti-pain (151/205; 73.7%) Table 1.

#### Knowledge score difference regarding dengue and chikungunya among HCWs in Moshi Municipality

**Fig. 1:**
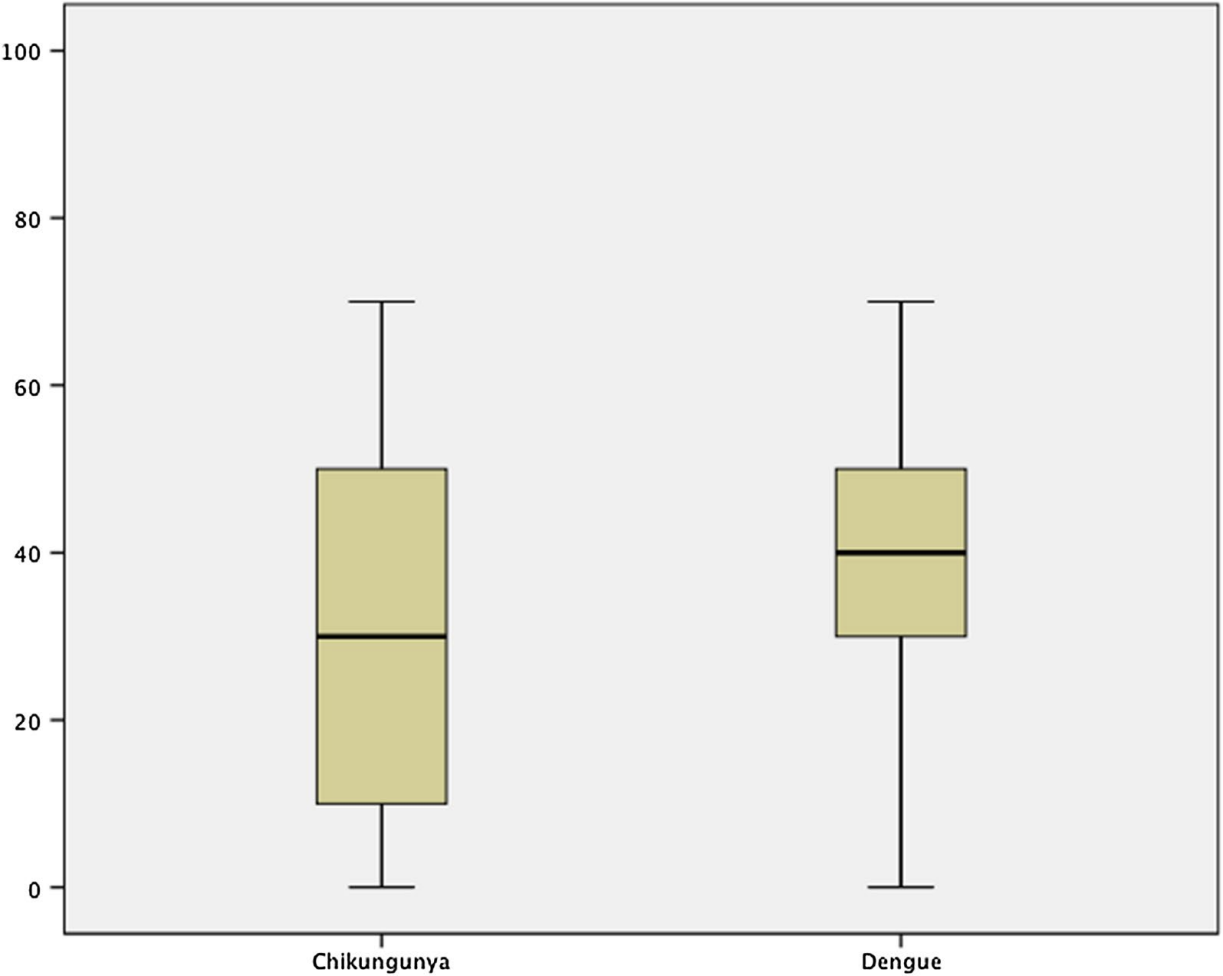
Mean knowledge regarding chikungunya and dengue fever

Among the HCWs, few participants (95/205; 46.3%) had good knowledge with regarding to chikungunya fever (knowledge score of 40 and above) with the mean knowledge score of (Mean ± SD) 31.7 ± 18.9. Also 152 (74.1%) had good dengue knowledge score with the mean knowledge score of (Mean ± SD) 43.6 ± 13.2 Fig. 1.

#### Diagnostic practices regarding chikungunya and dengue

HCWs were asked several questions concerning diagnostic practices of chikungunya and dengue in the health facilities. Most of HCWs (123/205; 60%) didn’t know if there is vaccine for chikungunya virus while (77/205 37.6%) reported absence of vaccine for chikungunya. Two (1.0%) reported that there diagnostic tools for chikungunya and almost all HCWs (195/205; 95.1%) reported that the absence of diagnostic tools for chikungunya virus lead to difficulties in managing the infection (Table 2).

**Table 2:** Diagnostic practices regarding chikungunya and dengue infection among healthcare workers (N = 205)

Variable	Chikungunya	Dengue
	n (%)	n (%)
*Is there a vaccine for (Chikungunya or Dengue infection)?*		
Yes	5 (2.5)	18 (8.8)
No	77 (37.6)	122 (59.5)
Don’t know	123 (60.0)	65 (31.7)
*Is there any tools for laboratory diagnosis of (Chikungunya or Dengue infection)?*		
Yes	80 (39.0)	105 (51.3)
No	24 (11.7)	46 (22.4)
Don’t know	101 (49.3)	54 (26.3)
*In your facility, do you have diagnostic tools for (Chikungunya or Dengue infection)?*		
Yes	2 (1.0)	3 (1.5)
No	203 (99.0)	202 (98.5)
*Absences diagnostic lead to difficulties in managing the infections*		
Yes	195 (95.1)	199 (97.0)
No	0 (0.0)	3 (1.5)
Don’t know	10 (4.9)	3 (1.5)
*Diagnostic method for (Chikungunya or Dengue infection)?*		
PCR	24 (11.5)	51 (24.9)
Rapid diagnostic test	32 (15.6)	47 (22.9)
ELISA	43 (21.0)	50 (24.4)
Don’t know	106 (51.7)	57 (27.8)
*How do you advice patients to prevent themselves against (Chikungunya or Dengue infection)?*		
Wearing long clothes at afternoon	43 (21.0)	56 (27.3)
Use of repellents	94 (45.9)	139 (67.8)
Use of blankets at night	11 (5.4)	5 (2.4)
Don’t know	57 (27.8)	5 (2.4)

*PCR* polymerase chain reaction, *ELISA* Enzyme Linked Immunosorbent Assay

One hundred and twenty-two of HCWs (59.5%) reported that there is no vaccine for dengue virus. One hundred and five (51.3%) reported that there are diagnostic tools for dengue and almost all HCWs (199/205; 97.0%) reported that the absence of diagnostic tools for dengue virus lead to difficulties in managing the infection (Table 2).

#### Analysis of the association between chikungunya knowledge score and Socio-demographic characteristics

The results of univariate analysis of the selected predictors and chikungunya or dengue knowledge score are shown in Additional file 1: Table S1. None of the selected predictors were associated with chikungunya or dengue knowledge.

### Discussion

This study aimed to assess knowledge and diagnostic practices regarding dengue and chikungunya among HCWs in Moshi Municipality. This study identified knowledge gaps among HCWs that should be targeted to improve the HCWs ability to practice and manage dengue and chikungunya infections.

For control and management of diseases a good knowledge about aetiology, mode of transmission, management and control is needed. This study found that the good knowledge regarding chikungunya was 46.3%. This level of knowledge was not satisfactory among HCWs. HCWs are expected to be knowledgeable about diseases as this knowledge could have been obtained in medical schools. Findings of this study was higher as compared with the study conducted in other District but the same Kilimanjaro region, which reported a overall knowledge to be 2.4% [20]. Slightly higher knowledge in this could be due to several reports of the disease in Tanzania [11, 13, 15, 17], and outbreak in nearby country where outbreaks of chikungunya had occurred at Lamu and Mombasa in 2004 [24]. A current study reported high dengue knowledge as compared by a study conducted by Kajeguka et al. [20]. The high knowledge of dengue among HCWs could be due to knowledge after the outbreak in the capital city Dar es Salaam in 2014 [25]. The outbreak created awareness to most of people in Tanzania. The same scenario has been reported in Singapore [26].

It is important to note that while majority of the HCWs were aware of dengue, a proportion of the respondents hold wrong notions about the cause, symptoms, transmissions and treatment of dengue. The HCWs didn’t know the treatments of the infections as most of them reported the treatments of infections as antiviral or antimalarial drugs. In a study of hospitalised febrile patients in northern Tanzania, most febrile illnesses like dengue and chikungunya were treated as malaria and some cases with antibacterial [13].

### Conclusion

There is insufficient knowledge regarding chikungunya fever among HCWs in Moshi Municipality. In view of this result, government and other non-government organizations should revisit the medical school curriculum especially in areas where vector borne diseases are prevalent. Refresher training of HCWs should be conducted regularly to improve their technical skills and build their capability.

## Limitation

Our results have some limitations. It is possible that some respondents might have provided desirable responses to some questions, since the study was a face to face interview. The study included a number of HCWs from Moshi Municipality, Small sample size which is limited only to HCWs from Moshi Municipality, hence finding are not nationally representative and cannot be generalized across the country.

## Additional file

**Additional file 1: Table S1.** Factors associated with good or poor knowledge regarding chikungunya and dengue infection.

## Abbreviations

HCW: Health Care Workers
WHO: World Health Organization
