# Supplementary material for: Healthcare workers knowledge and diagnostic practices: a need for dengue and chikungunya training in Moshi Municipality, Kilimanjaro Tanzania
Source: BMC Res Notes. 2019 Jan 18;12:43. doi: 10.1186/s13104-019-4074-x (PMC6339411; doi:10.1186/s13104-019-4074-x)
Supplement: Supplementary file 1 — Additional file 1: Table S1. Factors associated with good or poor knowledge regarding chikungunya and dengue infection. [file 13104_2019_4074_MOESM1_ESM.doc]

**Additional File 1, Table S1: Factors associated with good or poor knowledge regarding chikungunya and dengue infections**

| **Variable** | **Good n (%)** | **Poor n (%)** | **OR (95% CI)** | **p-value** |
| --- | --- | --- | --- | --- |
| **Chikungunya** | | | | |
| **Gender** |  |  |  |  |
| Male | 62(48.4) | 66(51.6) | 0.79 (0.45-1.41) | 0.4 |
| Female | 33(42.9 | 44(57.1) | 1 |  |
| **Age (in years)** | |  |  |  |
| <30 | 27(46.6) | 31(53.4) | 0.76 (0.24-2.42) | 0.6 |
| 30-40 | 51(48.6) | 54(51.4) | 0.70 (0.23-2.12) | 0.5 |
| 41-50 | 11(40.7) | 16(59.3) | 0.97 (0.26-3.51) | 0.9 |
| ≥51 | 6(40.0) | 9(60.0) | 1 |  |
| **Medical Role** | |  |  |  |
| Medical Doctor | 4 (36.4) | 7 (63.6) | 1.55 (0.32-7.36) | 0.5 |
| Ass. Med. Officer | 10 (43.5) | 13 (56.5) | 1.15 (0.32-4.07) | 0.8 |
| Clinical Officer | 29 (46.8) | 33 (53.2) | 1.01 (0.34-2.96) | 0.9 |
| Pharmacist | 7 (70.0) | 3 (30.0) | 0.38 (0.07-1.99) | 0.2 |
| Nurse | 37 (45.1) | 45 (54.9) | 1.08 (0.37-3.08) | 0.8 |
| Lab. Technician | 8 (47.1) | 9 (52.4) | 1 |  |
| **Working experience** | |  |  |  |
| <10 years | 72(46.8) | 82 (53.2) | 0.56 (0.23-1.40) | 0.2 |
| 10-15 years | 15 (55.6) | 12 (44.4) | 0.40 (0.12-1.24) | 0.1 |
| > 15 years | 8 (33.3) | 16 (66.7) | 1 |  |
| **Dengue** | | | | |
| **Gender** |  |  |  |  |
| Male | 98(76.6) | 30(23.4) | 0.72 (0.38-1.36) | 0.3 |
| Female | 54(70.1) | 23(29.9) | 1 |  |
| **Age (in years)** | |  |  |  |
| <30 | 46(79.3) | 12(20.7) | 1.69 (0.33-8.55) | 0.5 |
| 30-40 | 73(69.5) | 32(30.5) | 2.84 (0.60-13.36) | 0.1 |
| 41-50 | 20(74.1) | 7(25.9) | 2.27 (0.40-12.70) | 0.3 |
| ≥51 | 13(86.7) | 2(13.3) | 1 |  |
| **Medical Role** | |  |  |  |
| Medical Doctor | 8 (72.7) | 3 (27.3) | 1.21 (0.21-6.92) | 0.8 |
| Ass. Med. Officer | 17 (73.9) | 6 (26.1) | 1.14 (0.26-4.92) | 0.8 |
| Clinical Officer | 42 (67.7) | 20 (32.3) | 1.54 (0.49-5.35) | 0.4 |
| Pharmacist | 8 (80.0) | 2 (20.0) | 0.81 (0.12-5.49) | 0.8 |
| Nurse | 64 (78.0) | 18 (22.0) | 0.91 (0.26-3.14) | 0.8 |
| Lab. Technician | 13 (76.5) | 4 (23.5) | 1 |  |
| **Years of experience** | |  |  |  |
| < 10 years | 113 (73.4) | 41(26.6) | 1.37 (0.48-3.93) | 0.5 |
| 10-15 years | 20 (74.1) | 7 (25.9) | 1.33 (0.36-4.92) | 0.6 |
| >15 years | 19 (79.2) | 5 (20.8) | 1 |  |

OR= Crude Odds Ratio
